# Limb swelling consistent with posttraumatic lymphedema after closed upper and lower extremity fractures: A retrospective cohort study

**DOI:** 10.1371/journal.pone.0337756

**Published:** 2025-12-11

**Authors:** Felix Reinkemeier, Christoph Wallner, Marius Drysch, Sonja Verena Schmidt, Flemming Puscz, Maxi von Glinski, Thomas Armin Schildhauer, Marcus Lehnhardt, Björn Behr, Johannes Maximilian Wagner

**Affiliations:** 1 Department of Plastic Surgery and Hand Surgery, BG-University Hospital Bergmannsheil, Bochum, Germany; 2 Department of General and Trauma Surgery, BG-University Hospital Bergmannsheil, Bochum, Germany; 3 Department of Plastic, Reconstructive and Aesthetic Surgery, EVK Essen, Essen, Germany; Hiroshima University: Hiroshima Daigaku, JAPAN

## Abstract

**Introduction:**

Secondary lymphedema is a very common clinical issue with millions of patients suffering from pain, recurrent skin infections, and the constant need for a decongestive therapy. Well- established because of oncologic procedures, secondary lymphedema is also a well-known phenomenon after trauma. However, precise epidemiological data of posttraumatic lymphedema upon severe extremity injuries are rare.

**Methods:**

In the present study, we analyzed a patient cohort of 223 individuals who suffered closed fractures of the upper and lower extremity between 2016 and 2020. All of them simultaneously had a soft tissue injury, of 2nd and 3rd grade according to Tscherne classification. Typical symptoms of lymphedema were recorded in a retrospective cohort analysis through patient anamnesis and compared with documented clinical examination findings. Previous illnesses and trauma-specific characteristics were determined from patient files.

**Results:**

Of all patients, 36% showed symptoms of secondary lymphedema and 8,5% reported recurrent skin infections, indicating severe lymphedema. Furthermore, comparing patients with and without lymphedema, additional trauma-associated parameters, such as total number of surgeries, degree of soft tissue damage, localization of the fracture in lower extremity, related to lymphedema progress could be identified.

**Conclusion:**

According to these data, posttraumatic secondary lymphedema has even in closed fractures, especially of the lower extremity, a highly underestimated clinical prevalence. Further prospective studies are required to validate these findings, identify high-risk groups, and guide early prophylactic and therapeutic interventions.

## Introduction

Lymphedema is defined as a chronic inflammatory disease of the interstitium because of damage to the lymphatic drainage system. High hydrostatic pressure in the extremities causes fluid to shift into the interstitium. The high protein content of lymph further increases oncotic pressure, promoting fluid accumulation. This further increases the volume shift toward the interstitium. These two pathomechanisms exacerbate the clinical symptoms of lymphedema and often lead to chronicity of the disease [1]. Since lymph fluid is an ideal medium for the growth of bacteria, even minor injuries can be accompanied by recurrent soft tissue infections [1–3].

The diagnosis of lymphedema is usually based both on clinical criteria like volumetry and circumference measurement and a detailed medical history regarding predisposing factors, such as malignant disease or trauma. The leading symptom of manifest lymphedema is pronounced swelling with a significant increase in circumference and volume of the affected extremity. Although unilateral manifestation represents most secondary lymphedemas, bilateral manifestation is possible in principle. In addition, skin hardening and tissue fibrosis occur. Specific clinical signs of lymphedema include a pitting edema and a positive Kaposi-Stemmer sign. Further diagnostic measures include standard imaging diagnostics such as sonography, computed tomography and magnetic resonance imaging, as well as specific procedures such as near-infrared fluorescence lymphangiography and lymphoscintigraphy [4].

Based on the etiology, lymphedema can be divided into primary and secondary lymphedema [5–6]. Primary lymphedema can be caused by congenital hypoplasia or dysfunction of the lymphatic vessels, whereas the much more common secondary lymphedema is due to a lymphatic system damaged by various causes. Important factors are tumors and the frequently associated lymph node dissection, radiation, infections and trauma [1,7,8]. The most common cause of secondary lymphedema posed here is tumor resection. In particular, patients after cervical, axillary, or inguinal lymphadenectomy have a high risk of developing lymphedema [9].

Precise epidemiological data on secondary lymphedema are difficult to determine, but an incidence of 0.13–2% is assumed in industrialized countries [9]. The German Society for Lymphology reports 80,000 cases in Germany [9]. While the risk of developing lymphedema in oncological treatments is known, there is little data on the incidence and prevalence after trauma. Initial data indicate an increased risk after fractures of the long bones, with the development of lymphedema appearing to be associated with further complications [10].

In a first study with a small cohort, we could show that there is a prevalence up to 55% of posttraumatic lymphedema after open fractures of the lower extremity [11].

Based on these data, we continued the current study, which included a significantly larger patient cohort. Furthermore, the focus was now exclusively on patients with closed fractures. Patients with fractures of all extremities who underwent surgical treatment between 2016 and 2020 were included. Furthermore, this work aimed to identify risk factors, which are associated with an higher risk of developing secondary lymphedema.

## Materials and Methods

The study was conducted according to the guidelines of the Declaration of Helsinki and approved by the ethics committee of the Medical Faculty of the Ruhr University Bochum. A Written consent to conduct the study has been obtained. The study was conducted as a retrospective cohort study to collect data on the epidemiology, particularly the prevalence, of posttraumatic lymphedema following closed extremity fractures. Another aim of this study was to identify patient- and trauma-specific risk factors for the development of posttraumatic lymphedema. In total, 223 patients who sustained from closed long bone fractures of the upper and lower extremity with soft tissue injuries of grade 2 and 3 according to Tscherne were identified and contacted personally. Patients were asked about symptoms of lymphedema (e.g., prolonged swelling, pain, recurrent skin infections), and ongoing therapies (manual lymph drainage, compression garments). In addition, an evaluation of the clinical examination results recorded in the medical records and the outcome data of the patients was carried out.

Soft tissue injury of patients was classified according to ICD-10, adapted from Tscherne and Oestern classification.

The presence of lymphedema was defined by a combination of persistent swelling of the affected limb and the continuous need for decongestive therapy such as manual lymphatic drainage or compression treatment. In addition, the diagnosis required persistent swelling with ongoing therapy needs, supported by clinical findings such as pitting edema. Thus, the definition was based on both patient-reported history and objective clinical findings, in accordance with the consensus criteria of the International Society of Lymphology (ISL), which allow for a primarily clinical diagnosis without mandatory use of advanced imaging modalities.

### Patients

**Fig 1:**
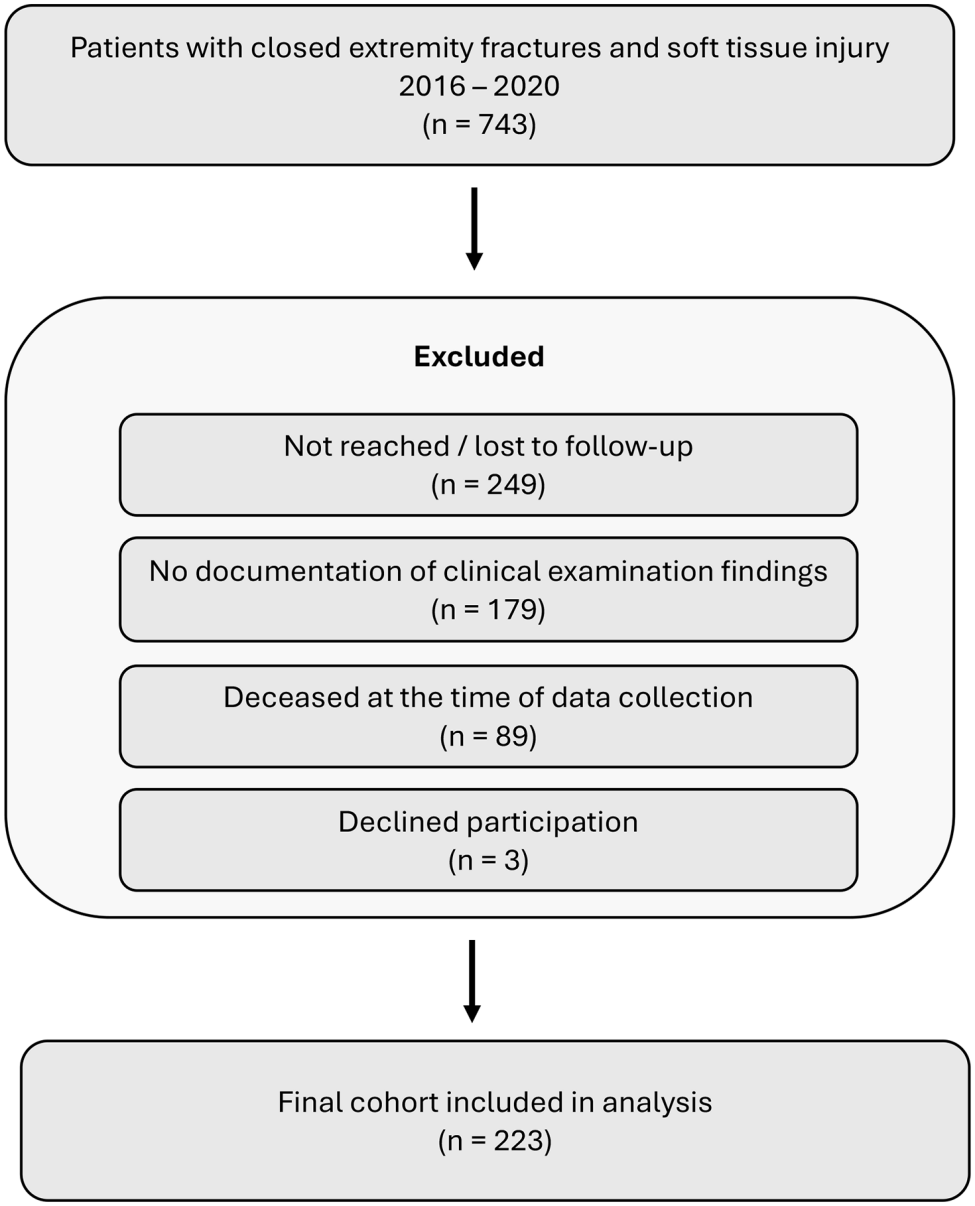
Flowchart of patient recruitment.

Inclusion criteria for this study were closed long bone fractures (femur, tibia, fibula, humerus, radius, ulna) of the upper or lower extremity combined with soft tissue injuries grade 2 and 3, according to Tscherne classification. Furthermore, all the included patients received at least one operation in the form of an open reduction and fixation. The patients were treated in a single institution between 2016 and 2020 (Fig 1). The data were accessed for research purposes between March and June 2024. The authors had no access to information that could identify individual participants during or after data collection.

### Statistics

Data are presented as means (range) or median. Patients were subdivided into two groups according to the occurrence of lymphedema and non-lymphedema. Pearson’s chi- square test and Fisher’s exact test was used for categorial variables (when expected value of any cell was below 5, Fisher’s exact test was used instead of chi-square test). For continuous variables, independent t-test was used. *p*-value below 0.05 was considered statistically significant.

In addition, we performed a multivariate logistic regression to identify independent predictors of posttraumatic lymphedema (dependent variable: lymphedema yes/no). Covariates were selected a priori based on clinical relevance and the reviewer’s recommendation and included: age category (<40 years, 40–65 years, > 65 years), sex (male/female), fracture location (femur, tibia, fibula, humerus, radius, ulna; indicator variables, > 1 possible per patient), severity of soft-tissue injury (3rd vs 2nd degree), multiple fractures, polytrauma, compartment syndrome, vascular/nerve injury, diabetes mellitus type 2, and BMI > 30 kg/m². Reference categories were <40 years, female sex, 2nd-degree soft-tissue injury, and absence of the respective condition. We report adjusted odds ratios (OR) with 95% confidence intervals (CI) and p-values. The model was fit by maximum likelihood on complete cases (listwise deletion). Analyses were performed in Python (statsmodels).

## Results

A total of 126 men and 97 women who suffered from closed bone fractures and 2–3° soft tissue injury between 2016 and 2020 were contacted personally and asked for ongoing symptoms such as swelling, pain and infections. Based on the patient’s remaining symptoms and subsequent comparison with the examination findings documented in the medical records, secondary lymphedema could be found in 81 (36.3%) of these 223 patients.

The mean age of all patients included was 51.9 years (18–87 years). In this regard, no significant difference could be detected between patients with (53.0 years) and patients without lymphedema (52.1 years, *p *= 0.862). Equivalent to our previous study on posttraumatic lymphedema in open fractures [11], a significantly increased number of patients in the lymphedema group were noted between 40 and 65 years (61.7% vs. 41.5%, *p *= 0.001). Older patients (>65 years) indeed did not have an increased risk for the development of posttraumatic lymphedema (Table 1).

**Table 1:** Patient characteristics.

	All patients n = 223 (%)	Patient with lymphedema n = 81 (36.3)	Patients without lymphedema n = 142 (63.7%)	p-value (comparison)
**Age mean (median)**	51.9 (18-87)	53.0 (18-87)	52.1 (18-83)	0.862
< 40	56 (25.1)	15 (18.5)	41 (28.9)	0.086
40–65	109 (48.9)	50 (61.7)	59 (41.5)	**0.001**
> 65	58 (26.0)	16 (19.8)	42 (29.6)	0.108
**Sex**				
Male	126 (56.5)	56 (69.1)	70 (49.3)	**0.004**
Female	97 (43.5)	25 (30.9)	72 (50.7)	
**Risk factors**				
Arterial hypertension	52 (23.3)	21 (25.9)	31 (21.8)	0.487
Diabetes mellitus type 2	14 (6.3)	9 (11.1)	5 (3.5)	**0.025**
Obesity (BMI > 30 kg/m2)	17 (7.6)	9 (11.1)	8 (5.6)	0.177
Smoking	51 (22.9)	18 (22.2)	33 (23.2)	0.862

With regard to pre-existing conditions, only diabetes mellitus was associated with an increased risk of developing posttraumatic lymphedema (11.1% vs. 3.5%, *p *= 0.025). The presence of obesity (BMI upon initial hospital stay) or arterial hypertension did not appear to be associated with a risk of posttraumatic lymphedema (Table 1). Consistent nicotine abuse since the trauma could not be identified as a significant risk factor for the development of posttraumatic lymphedema (22.2% vs. 23.2%).

Since patient-specific characteristics such as previous illnesses, gender and age do not appear to have a significant influence on the risk of developing posttraumatic lymphedema, an analysis of the trauma-specific characteristics was subsequently carried out.

Most of our patients suffered fractures of the lower extremity (57.0%), which was associated with a significantly increased risk of developing posttraumatic lymphedema compared to fractures of the upper extremities (p-value < 0.001) (Table 2). As a result, the diagnosis of lymphedema was due to a fracture in the lower extremity in more than three quarters of the cases (77.7% vs. 22.2%).

**Table 2:** Localization of fractures.

	All patients n = 223 (%)	Patient with lymphedema n = 81 (36.3)	Patients without lymphedema n = 142 (63.7%)	p-value (comparison)
**Fractured bone**				
Upper extremity	96 (43.0)	18 (22.2)	78 (54.9)	
Lower extremity	127 (57.0)	63 (77.7)	64 (45.1)	**<0.001**
Humerus	36 (16.1)	4 (4.9)	32 (22.5)	**0.001**
Radius	35 (15.7)	8 (9.9)	27 (19.0)	0.071
Ulna	20 (9.0)	8 (9.9)	12 (8.5)	0.720
Femur	22 (9.9)	5 (6.2)	17 (12.0)	0.163
Tibia	83 (37.2)	45 (55.5)	38 (26.8)	**<0.001**
Fibula	56 (25.1)	32 (39.5)	24 (16.9)	**<0.001**

Neither humerus fractures nor ulna and radius fractures were related to an increased risk of lymphedema (Table 2). Regarding humerus fractures, the risk was even lower. Interestingly, this is reversed in the lower extremities, as fractures in the distal area (lower leg) in particular are associated with a significantly increased risk, whereas femur fractures only lead to permanent lymphedema symptoms in exceptional cases.

Analyzing the injury mechanisms, we differentiated between contusion, traffic accident and fall. Analogous to the data of the previous study [11], a significantly increased number of patients in the lymphedema group (28.4%) sustained a traffic accident compared to trauma patients without lymphedema (14.8%, *p *= 0.014) (Table 3). While falls as a trauma mechanism did show a significantly lower risk for the development of lymphedema (p-value < 0.001), there was a significantly increased risk of lymphedema, especially for patients with a history of crush trauma (p-value < 0.001).

**Table 3:** Trauma characteristics.

	All patients n = 223 (%)	Patient with lymphedema n = 81 (36.3)	Patients without lymphedema n = 142 (63.7%)	p-value (comparison)
**Trauma Contusion**				
Traffic accident	44 (19.7)	23 (28.4)	21 (14.8)	**0.014**
Fall	152 (68.2)	43 (53.1)	109 (76.8)	**<0.001**
Trauma Contusion	34 (15.2)	24 (29.6)	10 (7.0)	**<0.001**
**Soft tissue injury**				
2^nd^ degree	191 (85.6)	57 (70.4)	134 (94.4)	
3^rd^ degree	32 (14.4)	24 (29.6)	8 (5.6)	**<0.001**
**Number of surgeries**				
1	90 (40.4)	19 (23.5)	71 (50.0)	**<0.001**
2	72 (32.3)	29 (35.8)	43 (30.3)	0.396
3	23 (10.3)	10 (12.3)	13 (9.2)	0.451
4	9 (4.0)	7 (8.6)	2 (1.4)	**0.008**
5 or more	18 (8.1)	15 (18.5)	3 (2.1)	**<0.001**
Multiple fractures	83 (37.2)	43 (53.1)	40 (28.2)	**<0.001**
Vascular/nerve damage	10 (4.5)	7 (8.6)	3 (2.1)	**0.023**
Polytrauma	19 (8.5)	12 (14.8)	7 (4.9)	**0.016**
Compartment syndrome	16 (7.2)	11 (13.6)	5 (3.5)	**0.005**
Soft tissue reconstruction	14 (6.3)	10 (12.3)	4 (2.8)	**0.005**

Interestingly, the extent of soft tissue damage also seemed to have an impact on secondary lymphedema formation. Third degree in comparison to second degree injuries were also associated with an increased risk of posttraumatic lymphedema (p < 0.001). Three quarters of all patients with third-degree soft tissue damage developed posttraumatic lymphedema.

In the following, we compared the total number of surgical interventions in our patient cohort, as this can be used as an indirect parameter for the severity of trauma and soft tissue damage. While only 23% of patients with lymphedema had undergone a single operation, this was the case in half of all patients without lymphedema (Table 3). A quarter of the lymphedema patients required four, five or more operations, whereas only 3.5% of patients without lymphedema had previously undergone this number of operations. The risk of developing posttraumatic lymphedema is therefore significantly reduced with one operation (p < 0.001), and significantly increased with four (p = 0.008) or more operations (p < 0.001).

In relation to the extent of the injury, a slight majority of 53.1% of lymphedema patients showed multiple fractures of the lower extremity, however, compared to non-lymphedema group (28.2%), statistical difference became evident (*p *< 0.001).

Interestingly, vascular or nerve damage and polytrauma patients were significantly enhanced in the lymphedema group (*p *= 0.023; *p *= 0.016). Finally, the occurrence of a compartment syndrome seemed to be related to lymphedema development in trauma patients (*p *= 0.005).

Reconstructive surgery was required in 12.3% of patients with posttraumatic lymphedema, whereas flap reconstruction and skin grafting were required in only 2.8% of patients without lymphedema (p = 0.005). The results are associated with divergent numbers of operations (Table 3).

Having analyzed trauma characteristics of the patients, we were further interested in complications and treatment as important indicators for development of posttraumatic lymphedema (Table 4).

**Table 4:** Clinical symptoms and therapy.

	All patients n = 223 (%)	Patient with lymphedema n = 81 (36.3)	Patients without lymphedema n = 142 (63.7%)	p-value (comparison)
Infection	19 (8.5)	12 (14.8)	7 (4.9)	**0.011**
Pain	91 (40.8)	55 (67.9)	36 (25.4)	**<0.001**
Compression therapy	39 (17.5)	31 (38.3)	8 (5.6)	**<0.001**

**Fig 2:**
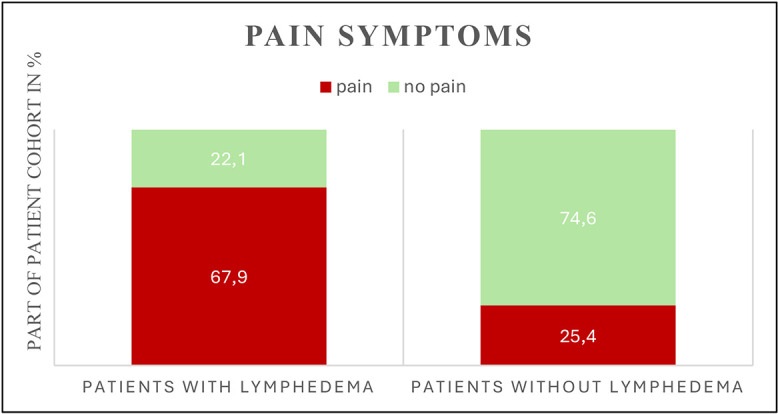
Proportion of patients reporting persistent pain, stratified by lymphedema status.

Almost two thirds of all patients with recurrent infections are among those with lymphedema (12 vs. 7 patients, p = 0.011). In addition, more than two thirds of all patients with lymphedema complained about persistent pain, whereas only about 25% of patients without lymphedema had corresponding symptoms (Fig 2). Compression therapy in the form of lymphatic drainage and wearing compression garments was necessary for almost 40% of lymphedema patients even years after trauma. In patients without lymphedema, however, this is an exception (5.6%, p < 0.001).

The clinical relevance is reflected on the one hand in the complication rate and on the other hand in the frequently persistent symptoms. The associated high therapeutic response is evident from the ongoing compression therapy in many patients with lymphedema.

### Multivariable analysis

Because several patient- and trauma-related factors were significantly associated with lymphedema in univariate analysis, we additionally performed a multivariate logistic regression to assess independent effects while adjusting for potential confounding (Table 5). The model included age categories (<40 years, 40–65 years, > 65 years), sex, fracture location, degree of soft tissue injury, multiple fractures, polytrauma, compartment syndrome, vascular/nerve injury, diabetes mellitus type 2, and BMI > 30 kg/m². In this adjusted analysis, the age group 40–65 years emerged as an independent risk factor for posttraumatic lymphedema (OR 2.48, 95% CI 1.10–5.62, p = 0.029). Likewise, third-degree soft tissue injury (OR 3.02, 95% CI 1.12–8.13, p = 0.029) and diabetes mellitus type 2 (OR 6.83, 95% CI 1.61–28.91, p = 0.009) were independently associated with increased risk. Conversely, humerus fractures were associated with a lower risk of lymphedema (OR 0.19, 95% CI 0.05–0.67, p = 0.010). Male sex showed a trend towards significance (OR 1.91, 95% CI 0.95–3.85, p = 0.071), as did multiple fractures and vascular/nerve injury. Other trauma-related parameters such as polytrauma or compartment syndrome did not reach statistical significance in the adjusted model. These findings suggest that while several trauma-related characteristics are strongly associated with posttraumatic lymphedema, their independent contribution is difficult to disentangle in a retrospective cohort due to overlapping effects and the co-occurrence of multiple severe injury patterns.

**Table 5:** Multivariate logistic regression analysis of independent risk factors for posttraumatic lymphedema.

	Odds Ratio	95% CI (low – high)	p-value
**Age mean (median)**			
40–65	2.48	1.10–5.62	**0.029**
> 65	1.50	0.57–3.97	0.413
**Sex**			
Male	1.91	0.95–3.85	0.071
**Risk factors**			
Diabetes mellitus type 2	6.83	1.61–28.91	**0.009**
Obesity (BMI > 30 kg/m2)	0.00	0.00 – ∞	1.000
**Fractured bone**			
Humerus	0.19	0.05–0.67	**0.010**
Radius	0.47	0.16–1.37	0.167
Ulna	0.87	0.23–3.30	0.834
Femur	0.63	0.18–2.27	0.479
Tibia	1.76	0.79–3.88	0.165
Fibula	1.09	0.48–2.50	0.839
**Soft tissue injury**			
3^rd^ degree	3.02	1.12–8.13	**0.029**
**Severity of trauma**			
Multiple fractures	1.96	0.93–4.14	0.079
Vascular/nerve damage	5.84	1.00–34.22	0.050
Polytrauma	1.12	0.28–4.42	0.877
Compartment syndrome	1.01	0.27–3.74	0.989

## Discussion

Although the occurrence of lymphedema after trauma was first described around 1960 [12,13] and although it is known that trauma can lead to the development of secondary posttraumatic lymphedema, data on its epidemiology are currently very scarce. Risk factors for the development of lymphedema after trauma were largely unknown until recently.

A first retrospective study on the epidemiology of posttraumatic lymphedema was published in 2021 [11]. This work showed a prevalence of 52% after open fractures of the lower extremities. Consequently, patients with open fractures seem to have a high risk for secondary lymphedema. Trauma-associated risk factors, rather than patient-specific characteristics, were identified as main contributing factors for secondary lymphedema formation. However, the number of patients examined was limited to open fractures. An analysis of closed fractures including fractures of the upper extremity has not yet been carried out.

In the present study, we analyzed 223 patients with closed fractures and 2° and 3° soft tissue injury of the upper or lower limb. All of them were treated by at least one operation at a single institution between 2016 and 2020. The prevalence of posttraumatic lymphedema is lower than the prevalence found in the preliminary study analyzing the epidemiology after open fractures. Nevertheless, more than one third of the analyzed patients showed typical symptoms of a secondary lymphedema of the injured limb. This prevalence is comparable to other patients who are at a high risk of secondary lymphedema. Garza et al reported a prevalence of approximately 20% in patients with axillary lymph node dissection after [4]. Returning to the introduction, in which tumor resection was stated to be the most common cause of secondary lymphedema, the development of secondary lymphedema in trauma patients appears to be associated with a similarly high prevalence. However, relevant literature providing epidemiological data is still rare.

In recent years different therapeutic approaches for treating posttraumatic lymphedema have been published. Pereira et al. performed soft tissue reconstructions in trauma patients using SCIP flaps, which included the transfer of lymph vessels [14,15]. This method successfully prevented the occurrence of posttraumatic lymphedema in a small cohort. Based on these findings, an approach for the surgical prophylaxis of lymphedema after trauma has been proposed. In addition to soft tissue reconstruction using SCIP-LV, this includes the performance of lymphovenous anastomoses [16].

As a further therapeutic measure, vascularized lymph node transfers were performed in patients with manifest posttraumatic lymphedema. Although successful treatment has so far only been carried out in very small patient cohorts, this appears to be a suitable treatment option for reducing symptoms and circumference in upper [17] as well as lower extremities [18].

In a systematic review by Dahl et al. it was shown that various surgical treatment procedures such as lymph axiality and interpositional flap transfer, vascularized lymph node transfers, lymphatic vessel free flaps, lymphovenous anastomoses (LVAs), and autologous lymphovenous transfers could improve symptoms [19]. Although the successful treatment of posttraumatic lymphedema by means of surgical interventions was again confirmed in a systematic review, the authors concluded that in the future the identification of patients at risk should be the priority to be able to initiate prophylactic measures at an early stage [20].

In our study, we were able to show that the occurrence of posttraumatic lymphedema is accompanied by long-term symptoms such as pain and recurrent infections. Furthermore, compression therapy must be continued even years after the trauma. A rare but very serious complication of a prolonged lymphedema, the occurrence of angiosarcoma, has been reported by Sandsmark as a consequence of posttraumatic lymphedema [21].

Current literature indicates that multiple surgical treatment approaches seem to be effective in reducing the burden of posttraumatic lymphedema, but these should be the prioritized in patients at high risk in order to avoid recurrent symptoms and complications. However, with the exception of our preliminary study [11], there are currently no data available to identify patients or risk factors.

After defining the prevalence of posttraumatic lymphedema in patients after closed long bone fractures and soft tissue damage, which is demonstrably at high risk, we were interested in further patient or trauma related characteristics that could be used to define high-risk groups.

In identifying possible risk factors for the development of posttraumatic lymphedema, patient-specific characteristics appeared to play only a minor role. The supposedly significant differences in the age group of 40 years to 65 years and in male group, we assume this result to be biased by an increased severity of trauma within this groups.

Our results suggest that trauma severity correlates with risk of post-traumatic lymphedema. One indirect parameter that can be used for this purpose is the extent of soft tissue damage. The presence of third-degree soft tissue damage was associated with a significantly increased risk (Table 3). These observations are consistent with data from oncological studies. Wu et al. investigated the epidemiology of secondary lymphedema after resection of soft tissue sarcomas of the extremities. Resection of a tumor on the medial thigh was associated with the highest risk of developing lymphedema over time [22].

Both the presence of a high degree of soft tissue damage and the consequences of severe trauma such as damage to functional structures, multiple bone fractures and polytrauma are associated with an increased risk of developing lymphedema. Since damage to the lymphatic system appears to occur more frequently when these factors are present, the severity of trauma must be identified as an overarching risk factor.

Another sensitive parameter which could be identified in the data analysis was the number of total surgeries. Only 21.1% requiring one surgery developed a lymphedema, while 80.3% of patients in the non-lymphedema had one or two surgical interventions. Since patients with relevant soft tissue trauma often require multiple surgeries, the total number of surgeries seems to be a sensitive parameter for trauma severity [11]. In conjunction with this, the need for plastic surgery for soft tissue reconstruction also seems to be associated with an increased risk. On the one hand, such operations are usually preceded by high-grade soft tissue trauma, and on the other hand, the intervention itself represents another surgical procedure, so that the total number of interventions also increases. In this context, the use of free flaps taking into account lymphatic drainage (LIFT) should be discussed [16 23].

After our first study investigating lymphedema prevalence after open fractures [11], this study provides supplementary data regarding the prevalence of secondary posttraumatic lymphedema after closed fractures in upper and lower extremity. Nevertheless, we need further prospective studies to validate these preliminary data. When interpretating the data, it should be considered that the classification of soft tissue damage was performed by different surgeons, which may lead to a potential bias. Further limitations of this work concern the retrospective assessment based on anamnestic data and the corresponding medical records.

In addition to these univariate associations, we performed a multivariate logistic regression to adjust for potential confounding factors. Interestingly, in this adjusted model only the age group 40–65 years remained independently associated with the occurrence of posttraumatic lymphedema. Male sex showed a trend towards significance but did not reach statistical significance, while other trauma-related variables such as multiple fractures, polytrauma, compartment syndrome, or third-degree soft tissue injury were no longer independently associated. Importantly, this does not contradict our univariate findings, which consistently indicated strong associations of these trauma-related factors with lymphedema. Rather, it highlights that these factors frequently occur together in patients with severe trauma and therefore show overlapping effects in multivariable models. Taken together, our results suggest that several trauma-related characteristics contribute substantially to the risk of posttraumatic lymphedema, but that their individual weight is difficult to disentangle in retrospective analyses. Larger prospective studies with standardized diagnostics are needed to confirm these preliminary findings and to better isolate independent predictors.

## Limitations

A major limitation of our study is the diagnostic definition of lymphedema, which did not include objective imaging such as lymphoscintigraphy or ICG lymphangiography. Another limitation is the absence of standardized volumetric measurements. This was inherent to the retrospective study design and limited us to clinical and anamnestic data. However, several aspects argue against misclassification as transient posttraumatic swelling. First, all patients sustained their fractures between 2016 and 2020, and data were collected in 2024, i.e., at least four years after the index trauma. This time interval strongly suggests a chronic rather than an acute or subacute swelling. Second, diagnosis was based not only on self-reported symptoms but also on documented clinical examination findings, including pitting edema, which is considered specific for lymphedema. Third, our approach is consistent with the International Society of Lymphology (ISL) consensus, which recognizes lymphedema primarily as a clinical diagnosis. The absence of standardized objective diagnostics remains a key limitation. Future prospective studies should incorporate limb volumetry or lymphoscintigraphy to improve diagnostic accuracy.

## Conclusions

In this retrospective exploratory cohort study, we observed a considerable prevalence of posttraumatic lymphedema even after closed fractures of the extremities with concomitant soft tissue injury. Trauma-specific rather than patient-specific factors appear central to secondary lymphedema development. Given the retrospective design, reliance on clinical rather than standardized objective diagnostic criteria, and potential biases in soft tissue classification, these results should be interpreted with caution and regarded as hypothesis-generating. Future prospective studies with standardized diagnostic methods and larger patient cohorts are required to validate these observations and to assess the potential of early prophylactic and therapeutic interventions.

## Supporting information

S1 File Data template. (XLSX)
